# COMParative Early Treatment Effectiveness between physical therapy and usual care for low back pain (COMPETE): study protocol for a randomized controlled trial

**DOI:** 10.1186/s13063-015-0959-8

**Published:** 2015-09-23

**Authors:** Daniel Rhon, Julie Fritz

**Affiliations:** Center for the Intrepid, Brooke Army Medical Center, 3551 Roger Brooke Drive, Fort Sam Houston, TX 78234 USA; Department of Physical Therapy, College of Health, University of Utah, 520 Wakara Way, Salt Lake City, Utah 84108 USA

## Abstract

**Background:**

Low back pain is among the leading causes of medical visits and lost duty days among members of the United States Armed Forces and represents the highest 5-year risk of permanent disability in the US Army. For certain elements of care, the timing may be just as important as the type of care. The purpose of this study is to assess the impact of the timing of access to a physical therapist by patients with low back pain, by looking at outcomes and low back pain-related healthcare utilization over a 1-year period.

**Methods/Design:**

This trial will be a two-arm pragmatic randomized clinical trial occurring at two different clinical sites in the Military Health System. We will assess outcomes and related downstream costs for patients who access physical therapy at the primary care level compared to those that receive usual care only. There will be 220 consecutive patients randomized to receive care in either group (early physical therapy or usual care only) for the first 4 weeks, and these patients will then be allowed to receive any additional care dictated by their primary care provider for the following year. The primary outcome measure is the Oswestry Disability Index. Secondary outcome measures are the Global Rating of Change, Patient Satisfaction and 1-year healthcare utilization. Follow-ups will occur at 4 weeks, 3 months and 1 year.

**Discussion:**

This trial takes a pragmatic approach to delivering care by enabling a usual care environment for managing low back pain, while also allowing immediate access to physical therapy. After the initial intervention, the patient’s primary provider can continue to manage the patient as he/she normally would in practice. The Military Health System Data Repository will capture all low back pain-related healthcare utilization that occurs in order to allow for a comparison between groups. Analysis from retrospective cohorts has shown improved outcomes and decreased costs for patients that received early versus late physical therapy, but this has yet to be shown in prospective trials.

**Trial registration:**

ClinicalTrials.Gov NCT01556581 initially on 14 March 2012.

**Electronic supplementary material:**

The online version of this article (doi:10.1186/s13063-015-0959-8) contains supplementary material, which is available to authorized users.

## Background

Low back pain is a priority healthcare condition due to its high prevalence and the economic healthcare burden it imposes on society. In fact, the World Health Organization has listed it in the top 10 conditions with the highest disease burden on society. The problem is even worse in the military where the stress and strain of combat add to the prevalence of this injury, making it one of the largest causes of attrition in soldiers both in combat and in the garrison [1–4]. Over the last decade, low back pain has been among the leading causes of medical visits and lost duty days among members of the US Armed Forces [5, 6] and represents the highest 5-year risk of permanent disability in the US Army [7]. This makes it an important target for clinically oriented research to improve management and reduce costs.

Physical therapy is a treatment option for low back pain. Although utilization of physical therapy is relatively low, 7 to 16 % of those that seek care do so for low back pain, [8] and this utilization is slowly increasing [9, 10]. Despite the increasing utilization of physical therapy and associated costs, there is surprisingly little information on the effectiveness of physical therapy as an early management strategy for patients with low back pain. Recent evidence suggests that the timing of physical therapy may be one of the more important variables related to prognosis because it is associated with improved healthcare costs downstream and decreased future healthcare utilization. Two recent studies found that early physical therapy (<14 days) was associated with significantly less healthcare utilization and costs for the 18- to 24-month period past the episode of back pain [11, 12]. A separate study compared an initial management strategy of either advanced imaging or physical therapy for low back pain and found that choosing advanced imaging over physical therapy as the initial strategy resulted in significantly greater overall 1-year healthcare costs related to back pain (average of $4,793 more per person) [13]. This means that initial care decisions made by healthcare providers about low back pain, such as early referral to physical therapy, can have a substantial impact on cost and long-term outcomes. The timing of interventions, such as physical therapy, is an area that needs further inquiry, and has the potential to influence referral recommendations and treatment approaches for low back pain. While the value of early physical therapy for low back pain has been shown in cohort studies, it has not been evaluated prospectively in a clinical trial.

The purpose of this study is to assess the impact of early physical therapy in patients seeking care for low back pain. We will assess outcomes and related downstream costs for patients seen in primary care that either are referred for physical therapy immediately or are managed via usual care pathways.

## Methods/Design

### Study design

This study will be a prospective multicenter randomized clinical trial with two arms, comparing the effectiveness of two strategies for managing patients with a recent onset of low back pain. One strategy will be usual care (UC) based on a stepped care approach. If patients do not improve with initial care options, then the next level of care is provided, *etcetera* (for example, nonsteroidal anti-inflammatory drugs (NSAIDS) and rest, progression to advanced imaging and/or specialty care referral). The other strategy will involve immediate Physical Therapy (PT). The study was designed following the standard protocol items for randomized trials (SPIRIT) guidelines for planning clinical trials [14]. The study will examine functional outcomes and subsequent 1-year healthcare utilization in each group. The primary difference between the strategies is the management in the first 4 weeks. The UC group will be managed with stepped care during this period, receiving advice, education, activity limitation guidance and medications if needed but no early physical therapy. The PT group will receive all the same care, but will also receive eight sessions of physical therapy guided by a treatment-based classification (TBC) approach during the first 4 weeks. The differences in care between the two interventions will occur primarily in the first 4 weeks. The approach will be pragmatic in order to compare the effects of the two management strategies under realistic clinical circumstances. Therefore, we will not attempt to balance provider contact time between groups over the first 4 weeks or control further treatment decisions made after the first 4 weeks. Assessors will be blinded to the treatment group.

### Subject selection

Subjects will be a sample of convenience made up of male or female service members on active duty between the ages of 18 and 60 years (or emancipated minors), seen at two large military medical center. Subjects will be given the option to enroll in the study in consecutive order, as they report for medical care at the primary care clinics and meet the inclusion criteria. Women comprise 15 % of all soldiers, and therefore, we expect our sample to have 15 to 20 female subjects accordingly.

#### Hypothesis

The following hypotheses will be tested:

1. Early intervention strategies utilizing physical therapy will result in greater improvements in function and disability in the long-term compared to a stepped usual care approach.
2. Early intervention strategies utilizing physical therapy will result in decreased use of other medical resources (medications, imaging, additional healthcare) in the long-term (1 year) compared to a stepped usual care approach.
3. Baseline psychosocial factors will be associated with changes in clinical outcomes within each treatment group.

### Recruitment and enrollment

Subjects will be recruited from two large U.S. Army Medical Centers from the regular pool of patients seeking medical care for low back pain. These patients will be approached and asked if they want to participate in the study. If they agree, they will then be consented and screened. Consent will be obtained from each participant. Ethical review and approval has been provided by the Western Region Medical Center Institutional Review Board at Madigan Army Medical Center.

### Randomization

Randomization will be conducted using a blocked randomization list (block size 2 or 4) generated prior to the study. Randomization assignments will be sealed in opaque envelopes prior to beginning the study. Opening the randomization envelope will occur after completion of the baseline examination and the advice and education intervention, to avoid bias in the delivery of this aspect of the intervention. Patients in both groups will be instructed to follow-up with their primary care provider as needed. Patients in the PT group will be scheduled to begin physical therapy within 3 days.

**Fig. 1:**
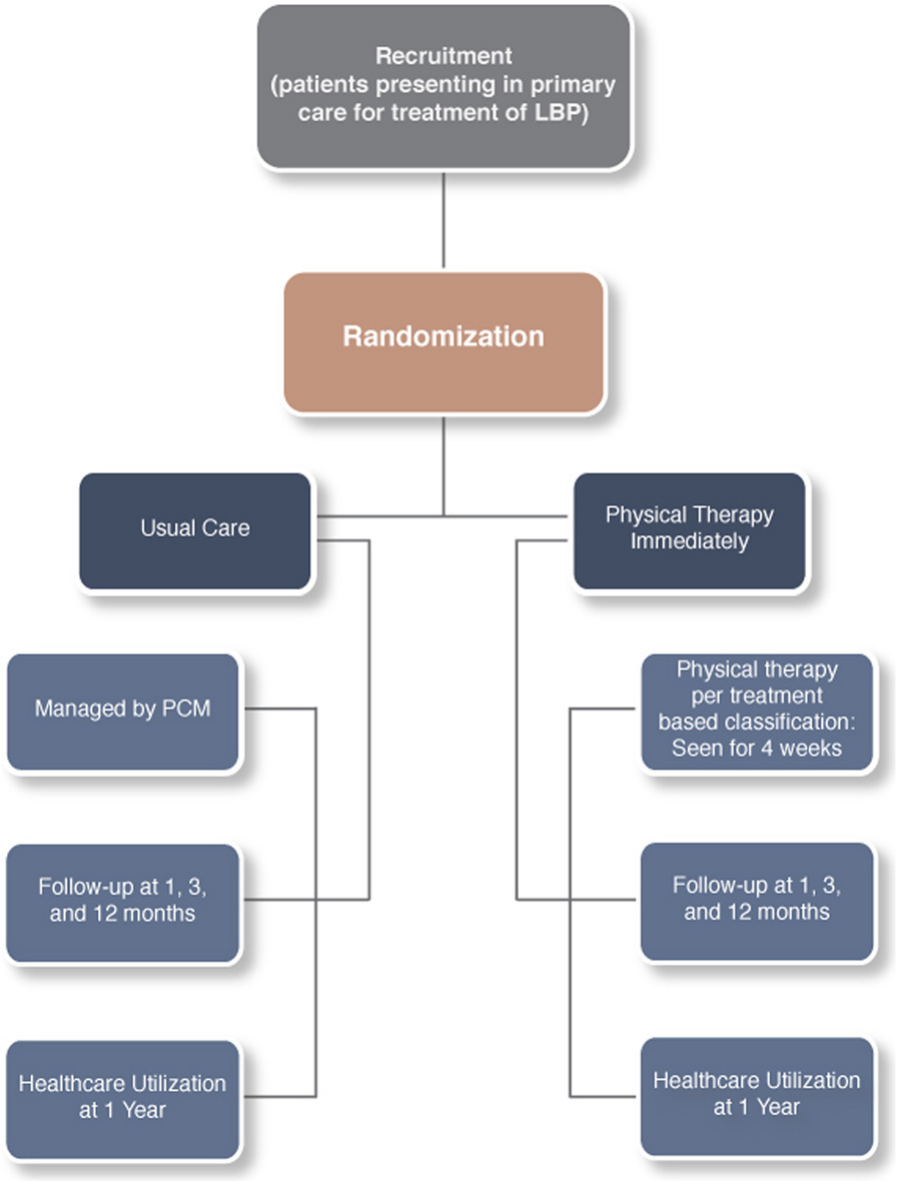
Study flow diagram. LBP = Low Back Pain, PCM = Primary Care Manager

We will record reasons for a subject dropping out of the study during any stage of the trial, and we will record all reasons for nonparticipation in the study to enable our ability to calculate an overall participation rate. The proposed flow of subjects through the study is depicted in Fig. 1.

### Treatment procedures

Those that meet the eligibility criteria will go through a baseline assessment and fill out the baseline surveys and self-report measures. All subjects will be evaluated and medically screened for red flags or other medical issues that would make them ineligible to receive physical therapy. After the baseline examination and all baseline measures are complete, all patients will receive a 20-minute educational session consistent with current evidence on self-management strategies (Additional file 1: Appendix 1). This will be considered the usual case. Every subject enrolled in the study will be given the option to receive a written excusal or modification of activity for up to 30 days and a 10-day prescription of NSAIDs and/or muscle relaxers if there are no medical contraindications (history of allergies to medications, GI upset, ulcers, GI pathology or renal pathology). Patients often receive this medication for back pain in primary care, and we do not want to encourage them to seek additional care outside the study in order to receive these medications if they have a strong desire for them, particularly if they are randomized to receive immediate physical therapy. Therefore, a reasonable dose will be offered to all subjects but will not be mandatory. Finally, the patient will be randomized either to receive only usual care or to see a physical therapist immediately for care.

#### Usual Care treatment group

Patients in the usual care (UC) group will receive the initial management outlined above. Consistent with a stepped care approach supported by practice guidelines, [15] no additional intervention will be provided to these patients, and they will be encouraged to follow-up for additional consultation on an as-needed basis if they feel they need additional care. At that time, consistent with practice guidelines, decisions on further treatments and/or referrals will be made by the patient’s primary care provider based on clinical judgment. All aspects of treatment provided to the UC group are part of the standard of care.

#### Physical Therapy (PT) treatment group

Patients in the PT group will receive the same initial management described above, and will then be referred to physical therapy within 48 hours. A maximum delay of 2 days should be inconsequential as typical recovery for back pain is 6 weeks, with a plateau at around 12 weeks [16]. The physical therapy treatment will be determined using a treatment-based classification approach, which has acceptable reliability [17]. This approach attempts to match a patient with an appropriate treatment based on their clinical presentation and relevant impairments. The three primary initial treatment approaches will use manual therapy (Additional file 2: Appendix 2), core strengthening (Additional file 2: Appendix 3), or an extension-oriented treatment approach (Additional file 2: Appendix 4). Typical of clinical practice within participating physical therapy clinics, the treating physical therapist will be allowed to modify the initial treatment strategy after 2 weeks of treatment if the patient demonstrates either substantial clinical improvement or worsening of symptoms. Substantial clinical improvement or worsening will be based on a repeat administration of the Oswestry Disability Index and patient self-report. Substantial improvement will be defined first by patient report and then verified objectively by administration of an Oswestry with improvement (that is, decrease in pain) of 50 % or more. Worsening will be identified first by patient report, and then defined as any deterioration (that is, increase) on a repeat Oswestry score. If either substantial improvement or deterioration is observed, the treating physical therapist may modify the initial treatment strategy. In other words, patients will also be progressed through the treatment program based on the clinical judgment of the treating physical therapist. Although they will start in one treatment group, they can progress to treatment in another group based on the directions of the physical therapist. Using the example above, if a patient has back pain with radicular leg symptoms that are successfully treated with the extension-oriented treatment approach after only five sessions and shows a subsequent improvement of 50 % on the Oswestry Disability Index, then the physical therapist may decide that the patient would benefit from core stabilization exercises and transition to this approach for the last three sessions, if impairments remain. All aspects of treatment provided to the PT group are part of the standard of care.

Patients in this group may receive a maximum of eight sessions over the 4-week treatment period (2 sessions per week). This is a typical treatment dosage for physical therapy for low back pain. At the end of this treatment period, patients are dismissed from care. If they feel that they need any further care, they are instructed to follow up with their primary care manager. All patients in this group will also be given an exercise program via handout (Additional file 3: Appendix 5) to perform at home.

### Data collection

The baseline examination will include a physical examination and completion of the self-report questionnaires listed here. The primary outcome variable is the Oswestry Disability Index. Secondary outcome variables include numeric pain rating, global rating of change, additional healthcare utilization and lost work/training time.

### Physical examination

A clinician will conduct a physical examination composed of the examination variables required to identify the patient’s treatment-based cluster. These variables include questions related to the distribution and duration of the patient’s symptoms, neural tension tests, measures of spinal range of motion including the effects of motion on the patient’s symptoms, assessment of spinal mobility and tests of trunk muscle function.

### Self-report questionnaires

After baseline assessment, the follow-up re-assessments will be performed at 4 weeks, 12 weeks and 1 year after the baseline examination. The assessor will be blinded to the patient’s treatment group assignment.

#### Oswestry Disability Index

The Oswestry Disability Questionnaire (OSW) was originally described by Fairbank et al. [18] as a condition-specific measure of functional status for patients with LBP. The OSW is a 10-item scale with higher numbers indicating greater disability. We will use the modified version that replaces the sex life item with an employment/homemaking item due to poor compliance with the former [19, 20]. The OSW is widely used in research on non-operative management of patients with LBP [21]. Our previous research has found the modified OSW to be used in this study to have high levels of test-retest reliability among stable patients (ICC = 0.90), good construct validity and responsiveness to change for patients with acute LBP, with a minimum clinically important difference (MCID) of six points for patients with acute LBP receiving physical therapy [19].

#### Numeric Pain Rating Scale

A 0 to 10 numeric pain rating scale (NPRS; ‘0’ indicating no pain, and ‘10’ indicating the worst imaginable pain) will be used to assess LBP intensity. Numeric pain scales are known to have excellent test-retest reliability [20]. Our previous research has found the NPRS to be responsive to change, with a minimum clinically important difference of two points among patients with acute LBP receiving physical therapy [22].

#### Fear Avoidance Belief Questionnaire

The Fear Avoidance Beliefs Questionnaire (FABQ) [23] will be used to measure patients’ beliefs about how physical activity and work may affect their LBP and perceived risk for re-injury. The FABQ contains two subscales: a seven-item work subscale (FABQW) and a four-item physical activity subscale (FABQPA). Test-retest reliability of the FABQ subscales is high [23, 24], and validity is supported by associations with disability and work loss in patients with acute and chronic LBP [25, 26]. Heightened fear-avoidance beliefs have been shown to be a risk factor for the development of chronic LBP following an acute episode [27, 28].

#### Pain Catastrophizing Scale

The Pain Catastrophizing Scale (PCS) is a 13-item patient-report scale developed to measure the extent to which people catastrophize in response to pain [29]. Each item is scored from 0 (‘not at all’) to 4 (‘all the time’). The PCS is reported as a total score, with higher scores indicating greater catastrophizing, and the scale is composed of three sub-scales: Rumination (four items, for example, ‘When I am in pain, I keep thinking about how badly I want the pain to stop’); Magnification (three items, for example, ‘When I am in pain, I become afraid that the pain will get worse’); and Helplessness (six items, for example, ‘When I am in pain, I feel I can’t go on’). The PCS has been shown to have high levels of internal consistency and construct validity [29, 30]. Pain catastrophizing has also been found to play a role in the transition from acute to chronic LBP [27].

#### Epworth Sleepiness Scale

The Epworth Sleepiness Scale (ESS) assessed the likelihood of falling asleep in eight scenarios commonly encountered in daily life [31]. The likelihood is ranked on a 0 to 3 scale, with 0 indicating that the individual would never doze and 3 indicating a high chance of dozing. It is one of the most commonly used self-assessment tools for measuring sleepiness [32]. Although not validated specifically in a population with low back pain, it has been shown to be a valid and reliable tool [33] for assessing sleep in other settings and populations [32, 34–37]. This may have a significant role in affecting outcomes of patients with low back pain as several preliminary studies have shown a correlation between chronic back pain and quality of sleep [38, 39]. Sleep deprivation is commonly seen in the active duty population with a high operational tempo.

#### Global Rating of Change

The patient will complete a global rating of change scale (GRC) at each follow-up. A 15-point scale is used as described by Jaeschke and colleagues [40], and this scale requires the patient to rate the degree of change in his or her condition from the beginning of treatment to the present. The midpoint of the scale is no change (0). Ratings from -1 to -7 represent varying degrees of a worsening of the patient’s condition, whereas rating from +1 to +7 represent varying degrees of improvement.

##### Patient satisfaction

Patient satisfaction with the care received for their LBP will be measured using a 10-item instrument that has been validated and found capable of distinguishing among three different dimensions of satisfaction (caring, information and treatment effectiveness) among patients with LBP attending primary care [41].

### Pain body diagram

A body diagram will be completed to identify the location and nature of symptoms [42]. Body diagrams can be used to reliably categorize the most distal extent of a patient’s symptoms [42, 43].

### Profile data

A profile is the official instrument used to restrict physical activities and work duties due to medical conditions. It is the only official document that allows a soldier to miss work or training. It provides a list of the activities that cannot be performed along with a duration of time that the restriction is in effect. It is what will be used to track work-loss days. This is recorded in number of days.

### Healthcare utilization

Finally, healthcare utilization data will be collected from the Military Health System Data Repository (MDR) database and will be confirmed via electronic medical record review. Healthcare utilization data will be used to determine any subsequent medical utilization related to low back pain and the economic impact of those injuries. These data will also allow a comparison of costs between the two treatment approaches used in this study.

### Data analysis

The data analysis for hypothesis number 1 will use separate analyses of covariance (ANCOVA) to compare the mean change in the primary (OSW) and secondary (NPRS, FABQ, PCS and GRC) outcomes from baseline to each follow-up assessment between groups while controlling for the baseline level of the outcome being analyzed to account for regression to the mean. We will assess the influence of baseline psychosocial measures (PCS and FABQ) on outcomes in both groups by introducing the scores as covariates into the ANCOVA in order to address hypothesis #3. For hypothesis #2, we will use chi-square tests and calculate odds ratios with confidence intervals for the specific healthcare utilization frequently sought by patients with LBP (MRI, injections, work loss as measured by the DA3329 limited duty profile).

We will make every effort to achieve complete data collection. For data that are missing, we will use multiple imputation (MI). Under the MI approach, baseline and follow-up factors beyond the variable being analyzed can be incorporated into the imputation model to account for dependence of the missing data mechanism on other measured factors, such as predictors of patient characteristics and prior scores. We will apply the method of data augmentation using Markov Chain Monte Carlo (MCMC) to generate imputed values. Four steps are involved: a) preparing the dataset for MI, which includes identifying all outcome variables likely to be involved in later planned or unplanned analyses, as well as likely predictors of missingness itself, evaluating distributions for normality and outliers and taking appropriate transformations to normality as needed; b) carrying out the MI, where variance and covariance estimates based on observed data are used to iteratively estimate maximum likelihood values for all participants, with multiple replacement values for each missing data point then being drawn randomly from the posterior distribution and perturbed with error; c) carrying out analyses identically on all versions of the imputed data; and d) combining tests and parameter estimates and adjusting for between-imputation variance to yield the final statistical results.

We will conduct all analyses based on intention-to-treat principles. This means that we will analyze all patients in the groups to which they were randomized regardless of compliance with the treatment protocols or follow-up assessments. Noncompliance with follow-up assessments will be handled using the MI approach outlined above. Per-protocol sensitivity analyses may be conducted if a high degree of treatment noncompliance is observed.

### Sample size estimation

Back pain and lower extremity injuries make up 44 % of all injuries for which soldiers seek care. Patients with back pain are seen on a daily basis in this healthcare system. Assuming at least 85 % of the patients complete the OSW at 12 weeks, enrollment of 110 subjects per group (total sample size 220 subjects) will provide at least 84 % power to detect a difference of seven points change in OSW at 12 weeks, assuming a standard deviation in the change in OSW of 16 points (treatment effect = 43.8 % of one standard deviation). The MCID for the OSW has been estimated at 6 points [19]. Previous work with patients with acute LBP indicate that these estimates of anticipated effect size and standard deviation are realistic, [44–46] and would be consistent with detecting an effect that is at least slightly in excess of the threshold for seeing significant change.

The natural history of recovery from back pain and our experience in prior studies suggest that differences tend to become less pronounced at 1 year than at 12 weeks. We believe 12 weeks is the appropriate time frame for the primary endpoint because it reflects the potential impact of treatment, and clinically, if patients are not experiencing improvement at this time point, they seek additional treatments. Thus, we are using this endpoint for the purposes of our power analysis rather than 1 year. The 4-week assessment will allow us to evaluate immediate post-treatment changes, and the 1-year assessment permits us to evaluate the potential long-term effects.

### Inclusion criteria

The inclusion criteria are as follows:

1. Military personnel on active duty and eligible for healthcare at a military treatment facility;
2. A primary complaint of low back pain defined as symptoms of pain and/or numbness between the 12^th^ rib and buttocks with or without symptoms into the leg(s), which, in the opinion of the provider, are originating from tissues of the lumbar region;
3. Duration of current episode of low back pain ≤ 90 days;
4. Age 18 to 60 years (or emancipated minors on active duty); and
5. Available for the following 4 weeks to complete a regimen of treatment.

### Exclusion Criteria

Exclusion criteria are as follows:

1. Oswestry Disability Index <20 % (prevent a ceiling effect).
2. History of receiving any medical care for this episode of low back pain within the last 3 months.
3. Prior surgery to the thoraco-lumbar spine or pelvis.
4. This episode of back pain is due to a traumatic fracture.
5. Pending a medical or physical evaluation board or discharge process, pending any litigation related to the condition, or planning on getting out of the military within the next 9 months.
6. Any “red flags” that would indicate a potentially serious condition or other significant disease process. These could include but not limited to cauda equina syndrome, large or rapidly progressing neurological deficit, fracture, cancer, ankylosing spondylitis, or other systemic disease.
7. Current episode occurred because of a motor vehicle accident.
8. Currently pregnant (or history of pregnancy in the previous 6 months).

## Discussion

There are challenges to implementing a clinical trial such as this. One of these is the nature of treatments that may potentially not be made available to patients, particularly those randomized to early physical therapy. Unless red flag screening indicates otherwise, imaging will not be ordered for patients. While this follows published clinical guidelines, and although outcomes are not shown to improve (and in some cases worsen) when advanced imaging is ordered, studies show that patient satisfaction is associated with higher rates of ordering of imaging [47]. Another challenge is the implementation of what is traditionally a specialty care service (physical therapy), but now is used at the primary care level. Physical therapy is typically a sequential service following primary care management, even though it is only utilized for 7-16 % of patients that present to primary care for back pain [11, 12].

It is feasible for patients with a new episode of LBP to receive physical therapy as part of an early management strategy [8]. However, physical therapy also contributes substantially to the healthcare costs associated with managing LBP [48, 49]. Along with the increasing utilization of physical therapy for LBP, are the corresponding costs that are also increasing [9, 10]. In the last 10 years, physical therapists in the US Army are now providing medical care further up on the front lines with the infantry soldiers as an organic asset to their unit. These therapists are in a prime position to facilitate early access to physical therapy interventions and have done so successfully in a combat-deployed setting [50, 51]. Considering that about 60 % of patients report recurrent symptoms, and 20 % report persistent disabling symptoms following an initial LBP episode [52, 53], research examining the most effective initial management strategies and the potential role of physical therapy is clearly needed. Suboptimal effectiveness of primary care management for patients with an acute episode of LBP can also lead to use of increasingly invasive and costly interventions, including injections, narcotic pain medications and surgical procedures [54–56]. In addition to the significant fourfold increase in prescription pain medication use in soldiers, other significant military healthcare costs are associated with the diagnostic management of these soldiers. In the current combat environment, advanced diagnostic imaging is not readily available [51]. Tests that would routinely be ordered in regular hospitals/clinics, when ordered in a combat setting, often require an evacuation out of the theater, incurring significant additional evacuation costs and time loss from duty. Especially for low back pain, many of these tests do not offer a valid prognosis or provide guidance for treatment and likely lead to an increase in overall medical costs [20, 57]. Reducing trends toward increased utilization and costs associated with prolonged episodes of LBP will require more effective early management strategies [58]. In a combat deployed setting, effective management strategies have the potential to decrease significantly the number of unnecessary evacuations out of the combat theater. The initial primary care encounter may therefore provide the best window of opportunity to improve outcomes and reduce costs related to low back pain.

## Trial status

The study is still actively recruiting subjects at the time of submission.

## Additional files

Additional file 1:
**Appendix 1.** Self-management Handout. (PDF 5448 kb) Additional file 2:
**Appendix 2.** Treatment-based Classification Approach - Manual Therapy. **Appendix 3.** Treatment-based Classification Approach - Core Strengthening. **Appendix 4.** Treatment-based Classification Approach - Extension Oriented Treatment Approach. (PDF 792 kb) Additional file 3:
**Appendix 5.** Home Exercise Program. (PDF 2337 kb)

## Abbreviations

FABQ: Fear Avoidance Belief Questionnaire
GRC: Global Rating of Change
LBP: low back pain
MDR: Military Health System Data Repository
MI: multiple imputation
MCID: minimum clinically important difference
MCMC: Markov Chain Monte Carlo
NPRS: Numerical Pain Rating Scale
OSW: Oswestry Disability Index
PCS: Pain Catastrophizing Scale
PT: physical therapy
SPIRIT: standard protocol items for randomized trials
TBC: treatment-based classification
UC: usual care
