# Supplementary material for: COMParative Early Treatment Effectiveness between physical therapy and usual care for low back pain (COMPETE): study protocol for a randomized controlled trial
Source: Trials. 2015 Sep 23;16:423. doi: 10.1186/s13063-015-0959-8 (PMC4581511; doi:10.1186/s13063-015-0959-8)
Supplement: Additional file 1: — Appendix 1. Self-management Handout. (PDF 5448 kb) [file 13063_2015_959_MOESM1_ESM.pdf]

REHABILITATION EXERCISES

Lumbar Spine

Hand Heel Rock

YouTube Video Demonstration: http://bit.ly/zpeGqv

(If this is a .pdf on your screen click on the link above. If this is a printout, type in the address above)

PURPOSE: The purpose of this exercise is to improve the mobility in your lower back

Exercise Procedures:

1. Come up to a quadruped positon on your hands and knees
2. Rock backwards so that your buttocks go towards your heels (almost sitting on heels) (B)
3. Then roll forwards past the starting position to a prone press-up (C)
4. Going all the way back and then forward equals one repetition
5. You will be alternating from flexing your back to extending your back.
6. This should not be a painful exercise

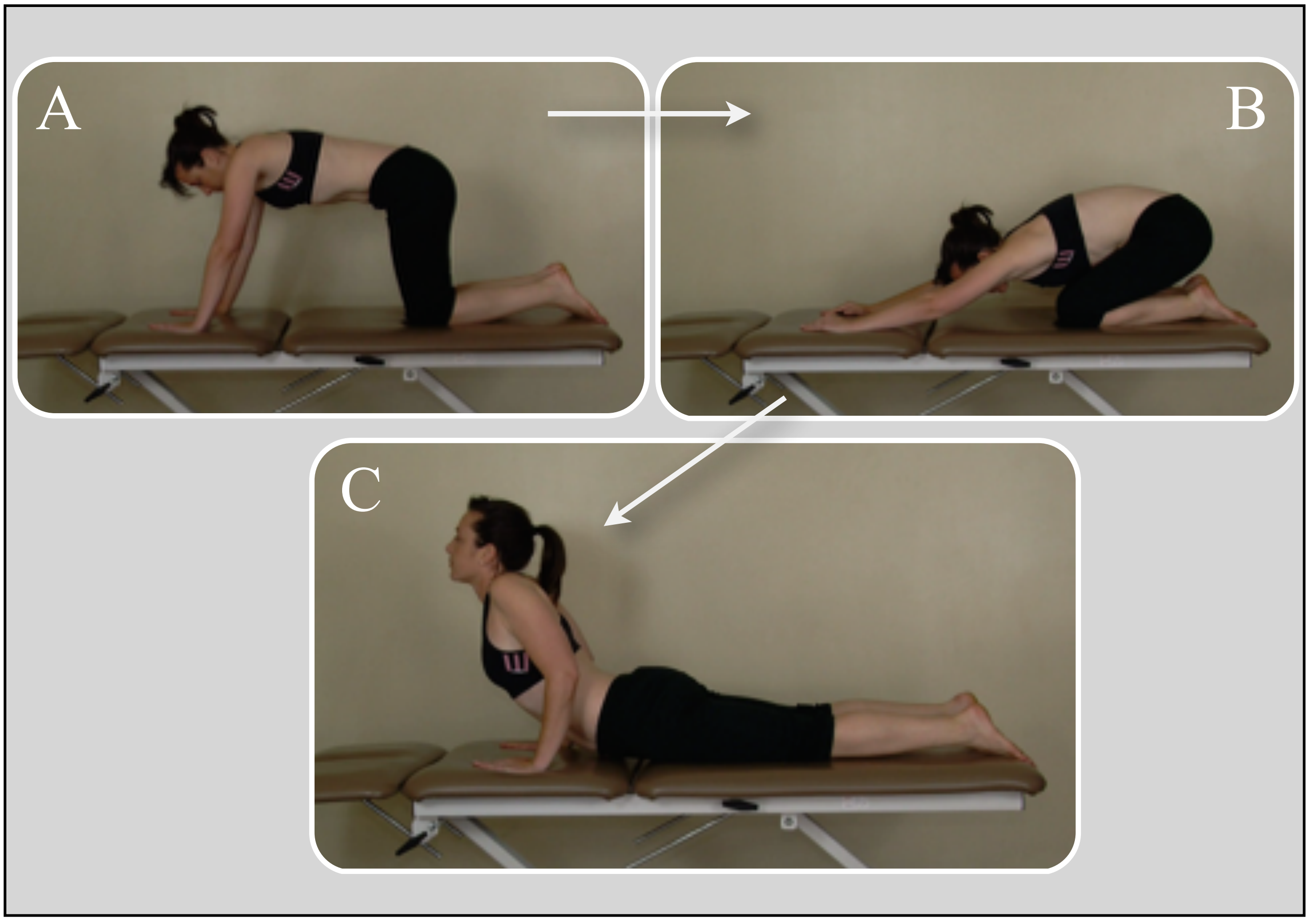

Dosage:

Perform 3 sets of 15 repetitions

How Often (per week)? The exercises should be performed twice a day

Common Mistakes to Avoid: If you move your hands back too far, you won't get as good of a stretch in your lower back. Try to find the place where you still feel a good stretch.

Images used with permission from Clinically Relevant Technologies, Seattle, WA

REHABILITATION EXERCISES
Lumbar Spine

Lumbar Rotations

YouTube Video Demonstration: **http://bit.ly/yE33oY**
(If this is a .pdf on your screen click on the link above. If this is a printout, type in the address above)

Exercise Procedures:
1. Lay on your side with the bottom leg slightly bent and the top leg bent up to about 60 degrees of hip flexion.
2. Place the hand of the top arm the side of your trunk (elbow is bent)
3. Place the hand of the bottom arm on top of the knee closest to you.
4. Stabilizing with the upper hand, push down on your knee in an oscillatory fashion to create a controlled rotation movement in your lower back on that side.

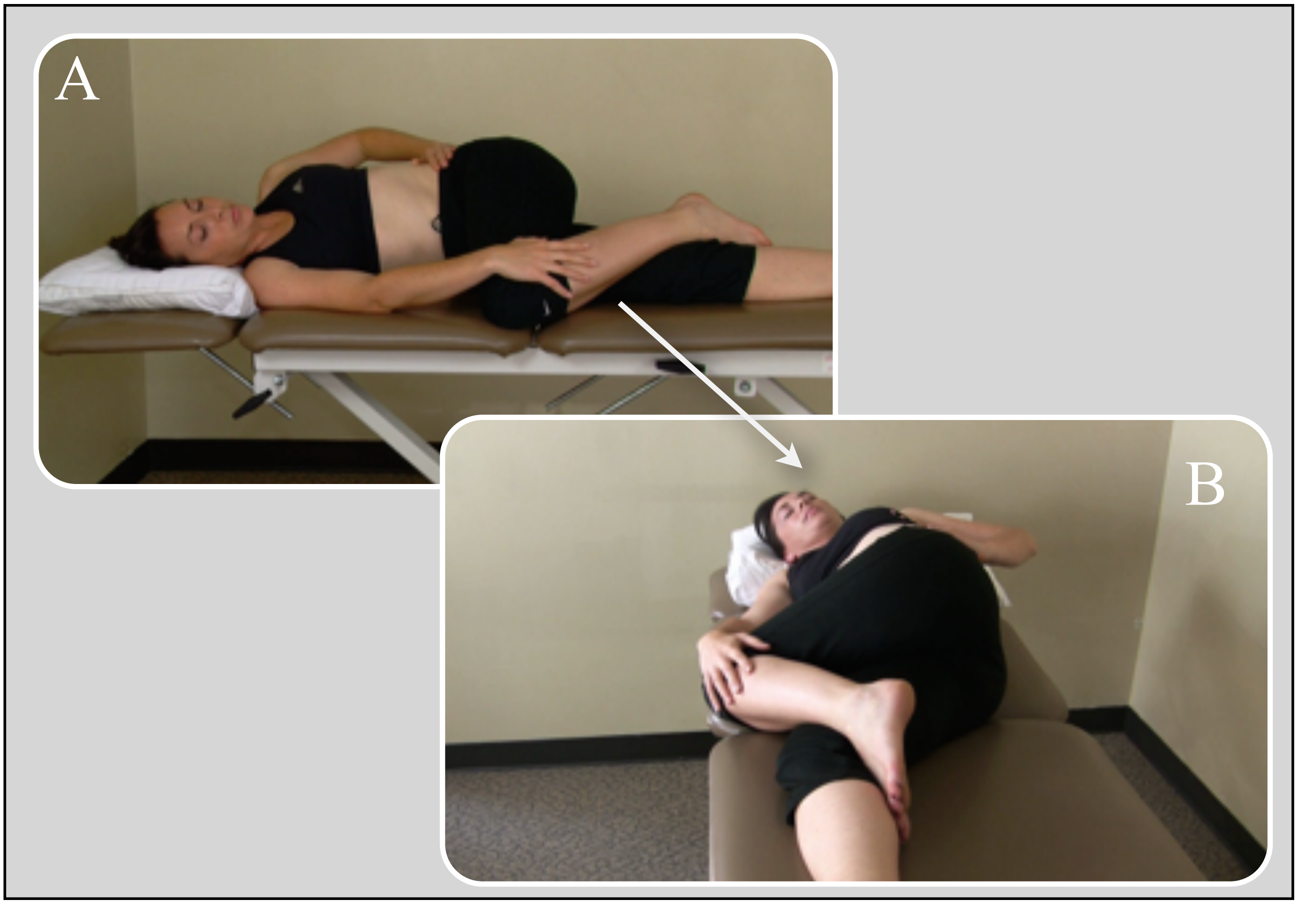

PURPOSE: The purpose of this test is to improve movement of the segments in your lower back

Dosage:
Hold for 3-5 seconds and perform 3 sets of 10 repetitions at the current level of progression you are on.
How Often (per week)? **Every 4-5 hours throughout every day**

Common Mistakes to Avoid: Try to keep your body aligned in straight line

Progression:

Images used with permission from Clinically Relevant Technologies, Seattle, WA

<u>REHABILITATION EXERCISES</u>
Lumbar Spine

Pelvic Tilts

YouTube Video Demonstration: **http://bit.ly/xTeYjw**
(If this is a .pdf on your screen click on the link above. If this is a printout, type in the address above)

PURPOSE: The purpose of this exercise is to obtain some mobility in the joints of your lower back (lumbar spine).

Exercise Procedures:
1. Lay flat on your back with your knees bent
2. Roll your pelvis forward (arch in your back - Figure B)
3. Roll your pelvis backward (buttocks roll up and just slightly off the table - Figure C)
4. Slowly return forward again (Figure B).
5. Continue the "tilting" or "rocking" of the pelvis for 3 full minutes.

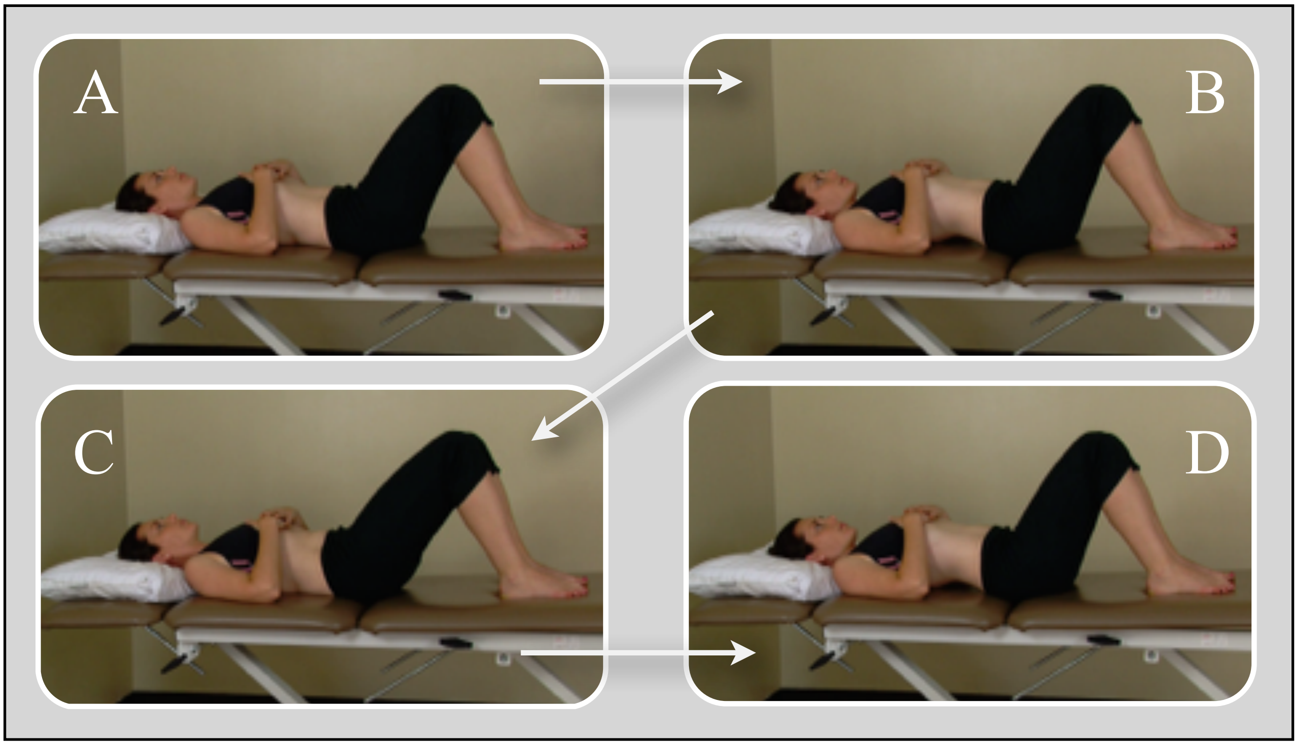

Dosage:
Perform for 3 minutes continuously taking approximately 2 seconds to go forward and 2 seconds to go back.
How Often (per week)? Every 4-5 hours during the day. Also perform at night before going to bed and first thing in the morning.

Common Mistakes to Avoid: Instead of a see-saw motion with your pelvis, try and move each individual segment of your low back and pelvis like the tracks of a bulldozer or tank, where one segment of your spine moves at a time.

Images used with permission from Clinically Relevant Technologies, Seattle, WA

REHABILITATION EXERCISES
Lumbar Spine (Low Back)

Extension Oriented Exercise Progression
YouTube Video Demonstration: http://bit.ly/zUhrv8

(If this is a .pdf on your screen click on the link. If this is a printout, type in the address above)

Purpose: The purpose of this test is to improve movement of the segments in your lower back

Exercise Procedures:

1. First attempt to lay out your stomach with your arms at your side. See if you are able to tolerate this for 5 minutes without the pain going further down into your leg.
2. Then progress by coming up on your elbows. See if you are able to tolerate this for 5 minutes without the pain going further down into your leg.
3. Then progress to doing this repeatedly for 10 repetitions (back and forth from Figure A to B).
4. Then progress to repeatedly extending back from a standing position (Figured C and D)

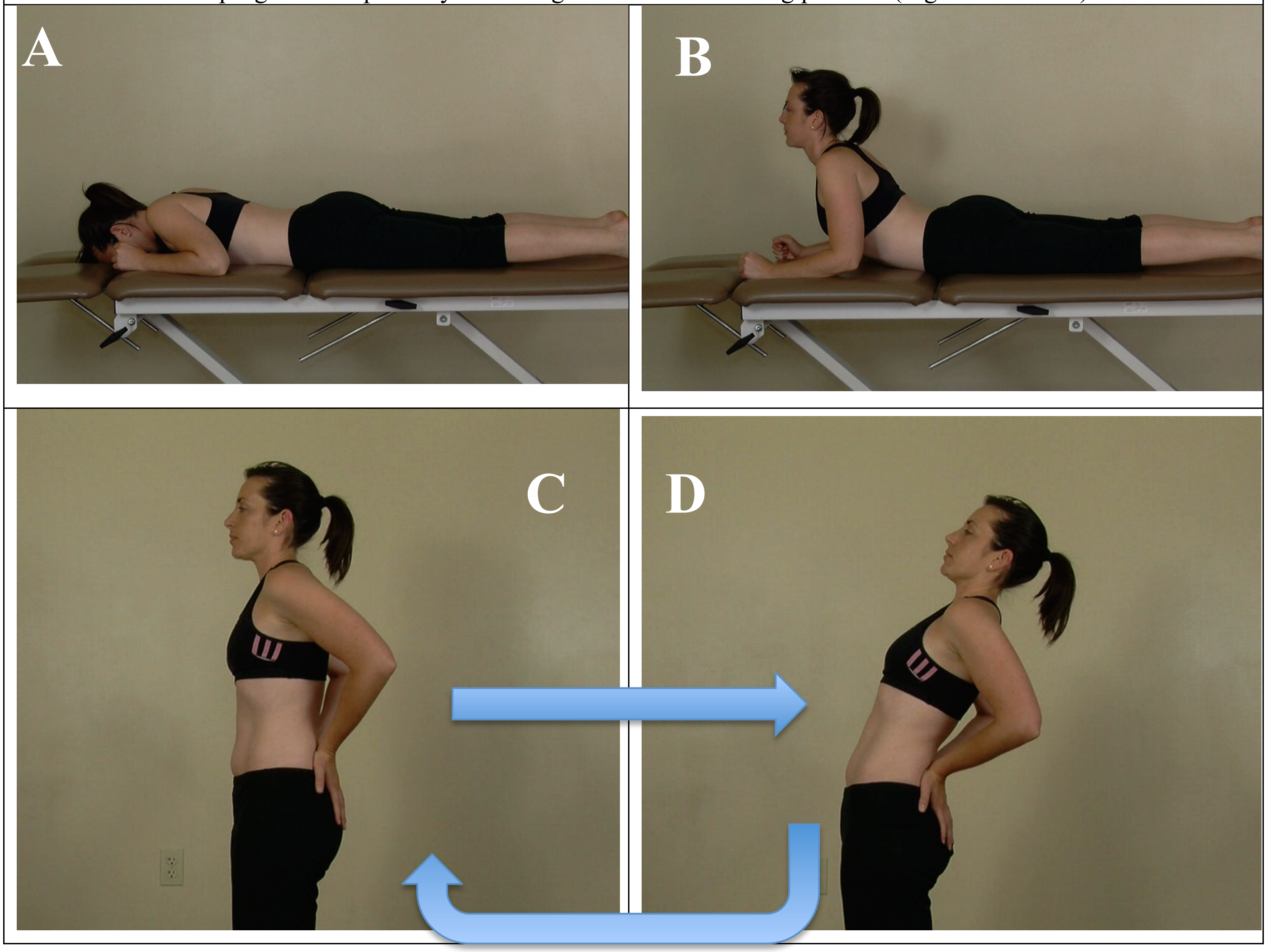

Dosage:

Perform _3 sets of_10_ repetitions of the standing exercise each time you do this. You should perform your exercises every 4-5 hours during the daytime.

Common Mistakes to Avoid: Try not to lift the pelvis up off the table.

Progression: Progress from laying on your stomach, to coming up on your elbows, to repeatedly coming up on your elbows, to repeatedly extending in a standing position.

Exercises to Reinforce Your Treatment

Images used with permission from Clinically Relevant Technologies, Seattle, WA

REHABILITATION EXERCISES

Lumbar Spine

Knee to Chest/ Hip Flexor Stretch

YouTube Video Demonstration: http://bit.ly/wzIVNV

(If this is a .pdf on your screen click on the link above. If this is a printout, type in the address above)

PURPOSE: The purpose of this exercise is to improve the flexibility of your hip muscles

Exercise Procedures:

1. Lay on your back with your knees bent and feet on the ground
2. Grab the front of one knee with both hands and pull it towards your chest (B)
3. Then straighten out your opposite leg (C)
4. You should feel a stretch in the anterior groin/quadriceps area of the thigh

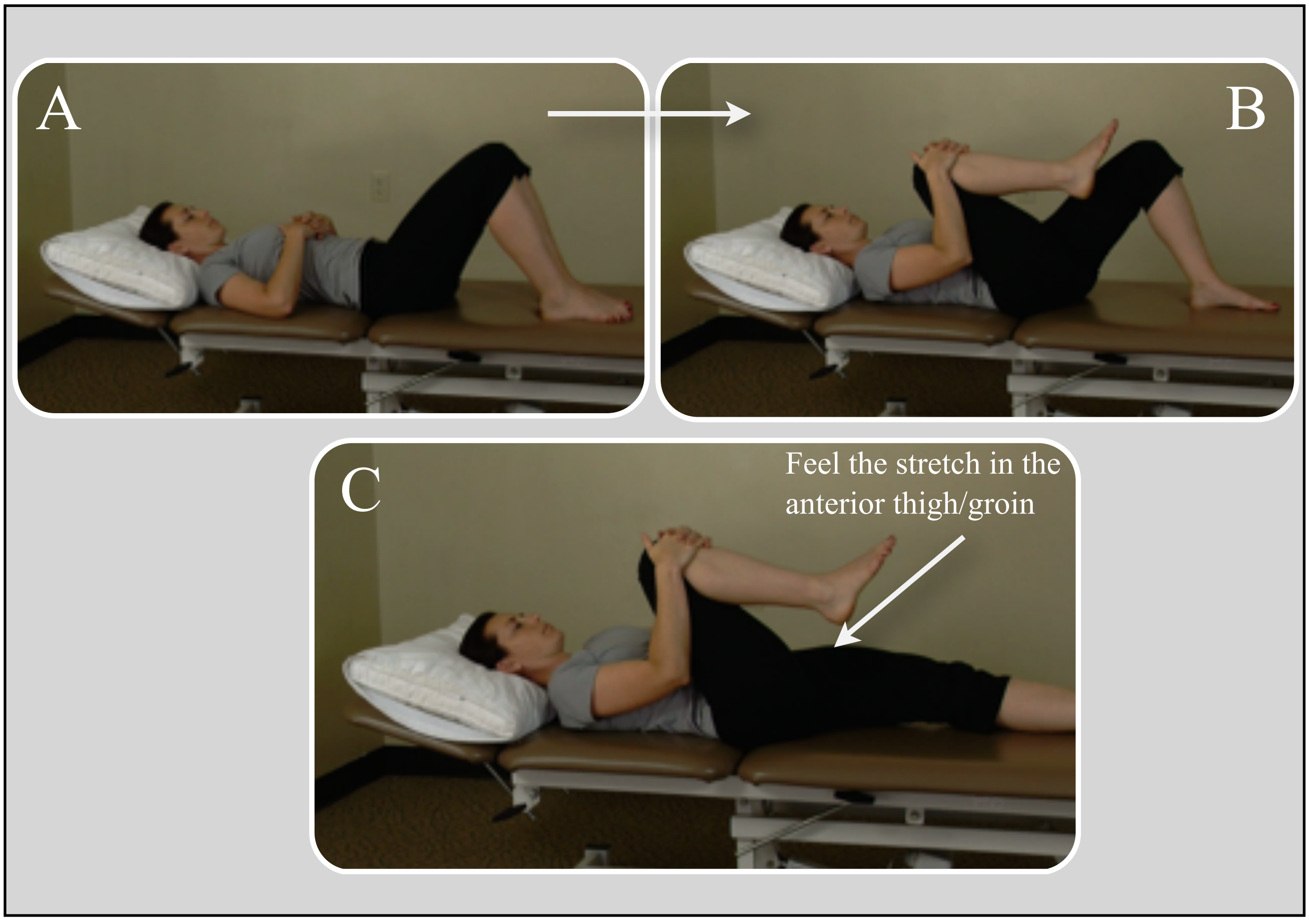

Dosage:

Hold the stretch for 30-60 seconds. Perform 3 sets of 10 repetitions

How Often (per week)? The exercises should be performed twice a day

Common Mistakes to Avoid: Try not to let the knee move too far away from your chest so as to maximize the stretch on the anterior hip region.

Images used with permission from Clinically Relevant Technologies, Seattle, WA

REHABILITATION EXERCISES
Lumbar Spine

ADIM - Hooklying Heel Slides

YouTube Video Demonstration: http://bit.ly/yZvxo6
(If this is a .pdf on your screen click on the link above. If this is a printout, type in the address above)

PURPOSE: The purpose of this exercise is to strengthen the core trunk muscles, specifically the transverse abdominus. Poor control of these muscles has been shown to have an association with certain types of low back pain.

Exercise Procedures:
1. Contract your abdominal muscles toward your spine
2. Hold this contraction while completing the rest of the steps
3. Keeping your knee bent, slowly raise one foot approximately 6-inches off the floor & hold for 3-5 seconds.
4. Slowly lower your foot to the starting position
5. Repeat the movement with the other leg

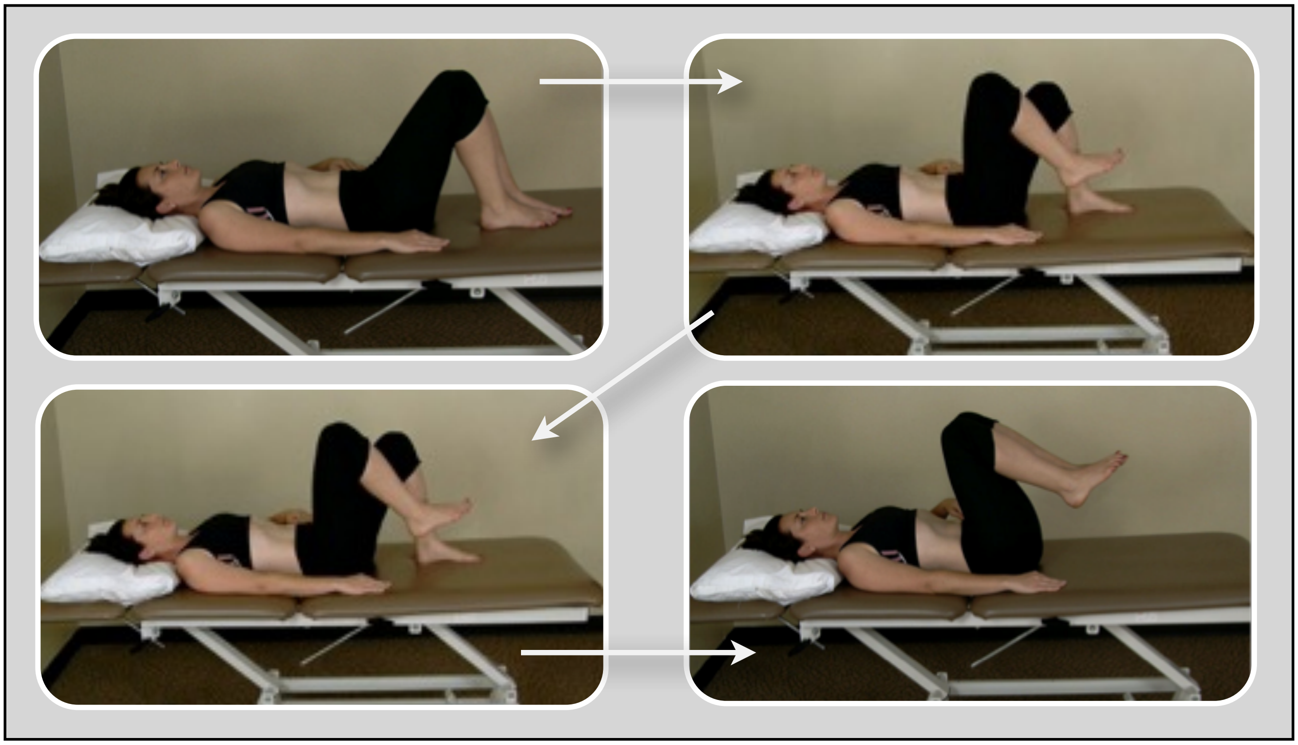

Dosage:
Hold for __3 to 5_____ seconds and repeat _____10_____ times
How Often (per week)? **Every 4-5 hours throughout the day**

Common Mistakes to Avoid: Not contracting the abdominal muscles while performing these exercises. You have to concentrate and be deliberate in contracting these muscles. Other mistakes include holding your breath, moving your head/neck/shoulders, and allowing your lower back to come off the table.

Progression: Progress from one leg at a time to both legs at a time

Images used with permission from Clinically Relevant Technologies, Seattle, WA

REHABILITATION EXERCISES
Lumbar Spine

ADIM Quadruped Exercises

YouTube Video Demonstration: http://bit.ly/zHb9pd
(If this is a .pdf on your screen click on the link above. If this is a printout, type in the address above)

PURPOSE: The purpose of this exercise is to strengthen the core trunk muscles.

Exercise Procedures:
1. Start on your hands on knees (4 points of contact), trying to keep your hips directly over your knees and your shoulders directly over your hands.
2. Perform the Abdominal Drawing in Maneuver (ADIM)
3. While performing ADIM, lift one arm up slowly, bringing it parallel to the ground (A)
4. Then bring the opposite leg up slowly until it is parallel to the ground (B)
5. Progress by lifting alternating arm and leg at the same time (C)
6. Progress by lifting same-side arm and leg at the same time.

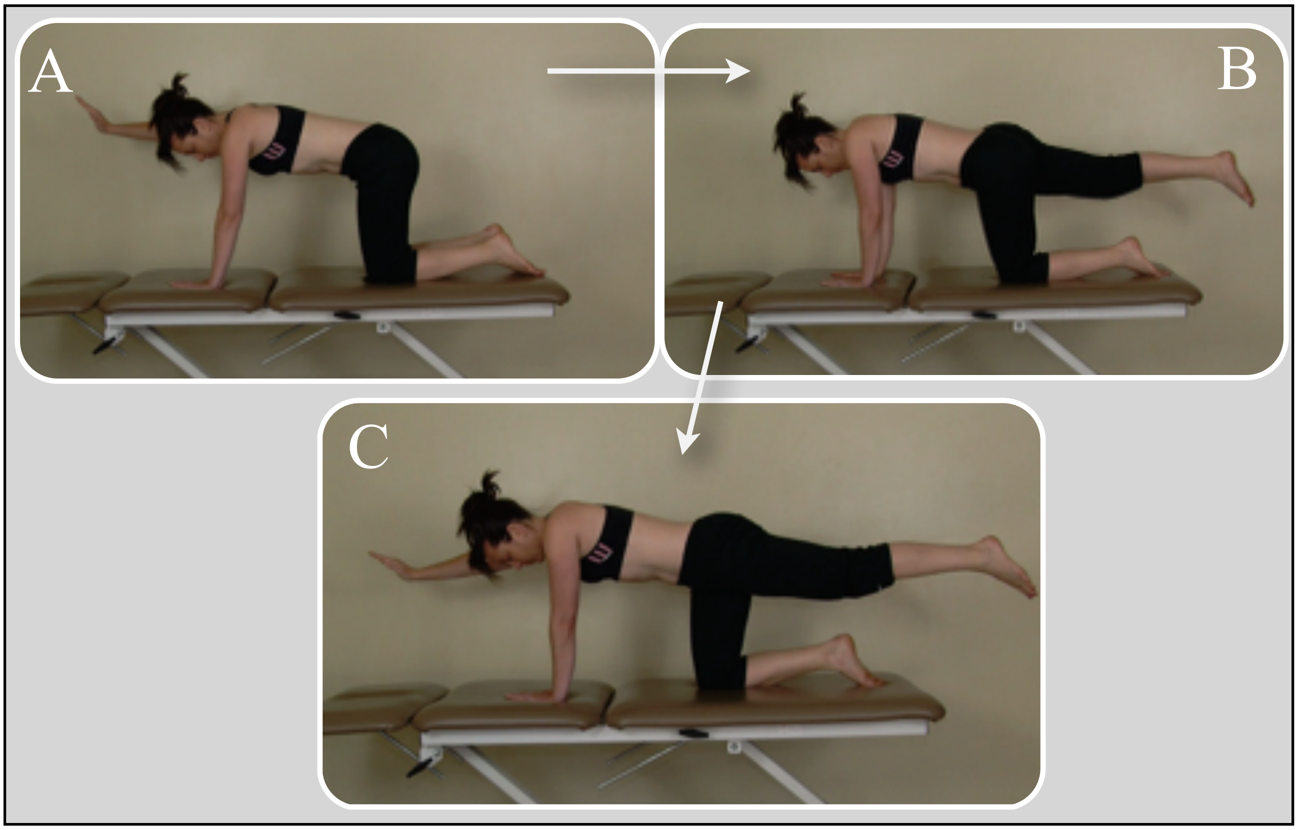

Poor control of these muscles has been shown to have an association with certain types of low back pain.

Dosage:
Hold for 3-5 seconds for each repetition and perform 3 sets of 10 repetitions at the current level of progression you are on.
How Often (per week)? **Every 4-5 hours throughout every day**

Common Mistakes to Avoid: It's very easy to lose the ADIM so you have to concentrate on doing it
Progression: Progress from single to alternating arm/leg to same side arm/leg

Images used with permission from Clinically Relevant Technologies, Seattle, WA

REHABILITATION EXERCISES
Lumbar Spine

Sideplank Core Exercises

YouTube Video Demonstration: http://bit.ly/zNYrQA
(If this is a .pdf on your screen click on the link above. If this is a printout, type in the address above)

PURPOSE: The purpose of this exercise is to strengthen the core trunk muscles. Poor control of these muscles has been shown to have an association with certain types of low back pain.

Exercise Procedures:
1. Start laying on your side with your elbow, hips, knees, and legs as points of contact.
2. Raise your hips off the table to make a straight line between your shoulder and your knee (B)
3. Hold the position for 3-5 seconds and then return to starting position (A)
4. Slowly lower your foot to the starting position
5. Repeat the movement with the other leg

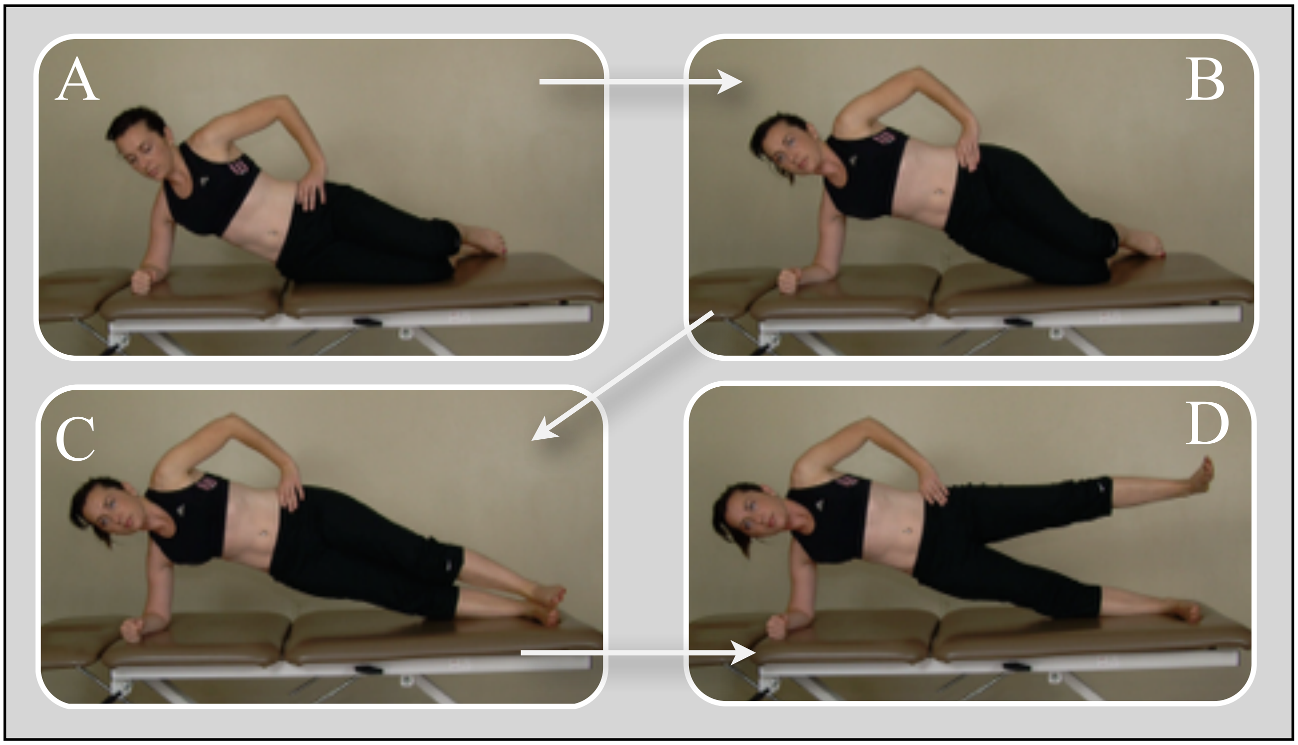

Dosage:
Hold for 3-5 seconds and perform 3 sets of 10 repetitions at the current level of progression you are on.
How Often (per week)? **Every 4-5 hours throughout every day**

Common Mistakes to Avoid: Try to keep your body aligned in straight line

Progression: Progress from having your knees as the point of contact (Figure A), to having your foot/ankle as the point of contact (Figure C) to then "scissor-kicking" your legs in the air after raising your hips off the table (Figure D).

Images used with permission from Clinically Relevant Technologies, Seattle, WA

REHABILITATION EXERCISES

Lumbar Spine

Bridging Progression

YouTube Video Demonstration: **http://bit.ly/AxhUnU**

(If this is a .pdf on your screen click on the link above. If this is a printout, type in the address above)

PURPOSE: The purpose of this exercise is to strengthen your trunk and core muscles

Exercise Procedures:

1. Lay flat on your back with your knees bent, with arms at your side
2. Tighten your gluteal muscles and push your pelvis up into a "bridge" position (B)
3. Progress by moving your arms from your side to placing them on your chest/stomach (E-F)
4. Progress by lifting one leg up off the table when bridging (C)
5. Progress by lifting one leg up off the table and extending your knee fully (D)
6. Progress by decreasing the stability of your **feet anchor by adding a pillow, bolster, or exercise ball (F)**

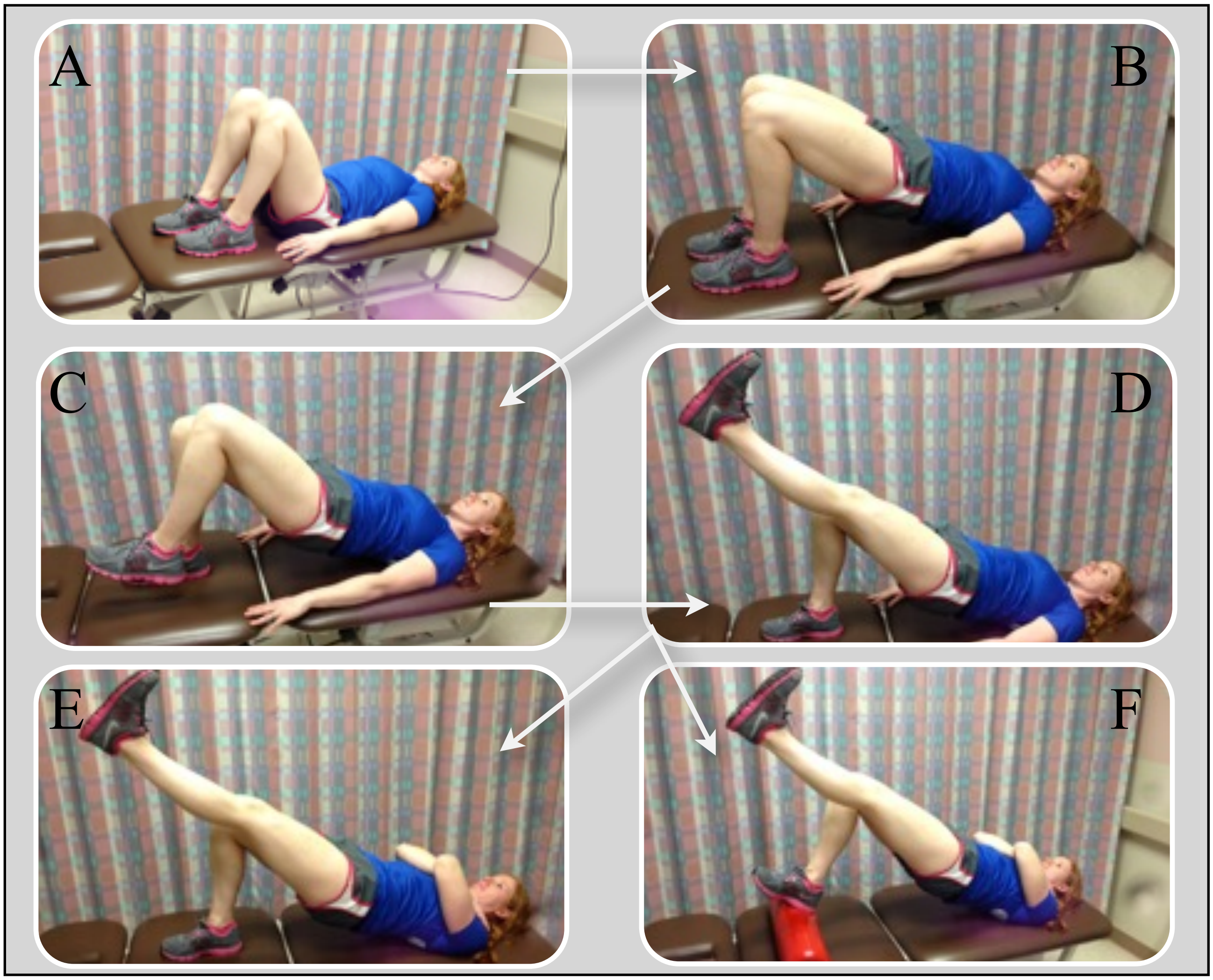

Dosage:

Hold each position for 5-10 seconds. Perform 3 sets of 10 repetitions

How Often (per week)? The exercises should be performed twice a day

Common Mistakes to Avoid: If your hamstrings begin to cramp, try to focus more on deliberately contracting your gluteal muscles as you push up.

Images used with permission from Clinically Relevant Technologies, Seattle, WA
