# Supplementary material for: COMParative Early Treatment Effectiveness between physical therapy and usual care for low back pain (COMPETE): study protocol for a randomized controlled trial
Source: Trials. 2015 Sep 23;16:423. doi: 10.1186/s13063-015-0959-8 (PMC4581511; doi:10.1186/s13063-015-0959-8)
Supplement: Additional file 2: — Appendix 2. Treatment-based Classification Approach - Manual Therapy. Appendix 3. Treatment-based Classification Approach - Core Strengthening. Appendix 4. Treatment-based Classification Approach - Extension Oriented Treatment Approach. (PDF 792 kb) [file 13063_2015_959_MOESM2_ESM.zip › additional file 2/13063_2015_959_add2.pdf]

Manual Therapy Treatment Protocol

COMPETE Low Back Pain Trial

This following is the treatment protocol for those receiving manual therapy to include spinal manipulation. A summary of the frequency of treatment is outlined below:

	Week 1	Week 2	Week 3	Week 4
**Manual Techniques and Supporting Exercises (8 sessions)***	2 sessions	2 sessions	2 sessions	2 sessions

* Subject in initially placed in other groups, may progress to the core strengthening/stability group during this 4-week period based on clinician judgment and response to manual therapy or EOTA.

The subjects in this group will receive a manual therapy intervention consistent with the evidence in patients that meet specific criteria when present, and based on impairments found during the exam in the rest of patients. Progression plan during the 4 week period of treatment will be based on response of patient to manual therapy based on clinician judgment, and may include progression of manual techniques and/or transition to the core strengthening class. Patient should also receive education as outlined below:

1. Education Component:
The clinician should review the education component on the patient's exercise handout. Key messages to emphasize should include:

- Frequency of the exercises
- Encourage to perform activities even if there is some slight discomfort.
- Encourage patient to stay active

2) Manual Therapy Component:
The treatment received by patients in this group will include at a minimum the lumbo-pelvic supine spine manipulation if symptoms have been present < 16 days and do not extend below the knee, unless there are significant contraindications present. Other manual techniques and progression for patients with other symptoms, and for progression of treatment in patients that do receive the lumbo-pelvic manipulation, will be documented by the clinician. The clinicians will use an impairment-based approach to target related impairments in the thoracic and lumbar spine and the hip and pelvis. The dosing and frequency of these other techniques will be documented by the treating clinician.

3) Manual Therapy Supporting Exercises Component:
Exercises: These exercises help support the manual interventions provided by the treating clinician, and help promote mobility and flexibility to reinforce the specific manual interventions given.

Exercise	Goal
1. Pelvic Tilts	3 minute repetition; 2-3x/day
2. Self-Rotations	30 second repetitions; 4-5x/day
3. Prone press-up progression	3x10 repectitions; 2-3x/day
4. Knee to chest	3x10 repectitions; 2-3x/day

<u>HEP:</u> Patients will be instructed to perform the exercises they are given every day according to the dosage for each exercise listed above.

Manual Physical Therapy Techniques

COMPETE Low Back Pain Trial

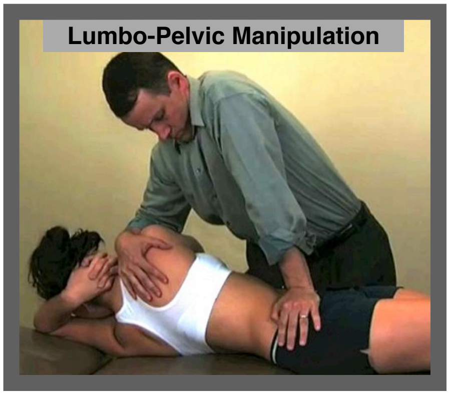
Lumbo-Pelvic Manipulation

Clinician Position:
Maximally sidebends patient away (C-shape), crosses legs and supports trunk to maintain position.
Technique Description:
Rotate trunk towards clinician while keeping C-shape. Provide thrust in posterior and caudal direction

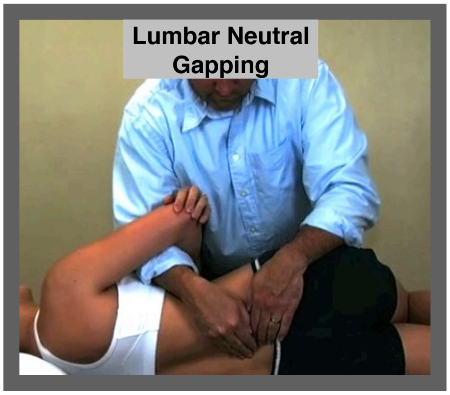
Lumbar Neutral Gapping

Clinician Position:
Stand facing patient - lean over so trunk is directly over the patient.
Technique Description:
Find intended segment. Log roll patient toward you. Use forearm of distal arm to induce an HVLA thrust in an anterior direction.

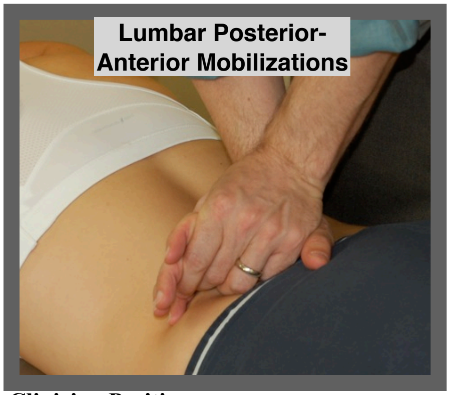
Lumbar Posterior-Anterior Mobilizations

Clinician Position:
Stand over patient with arms perpendicular to patients spine. Find intended segment.
Technique Description:
Apply a graded mobilization in the anterior direction either centrally at the spinous process or unilaterally at the articular pillar

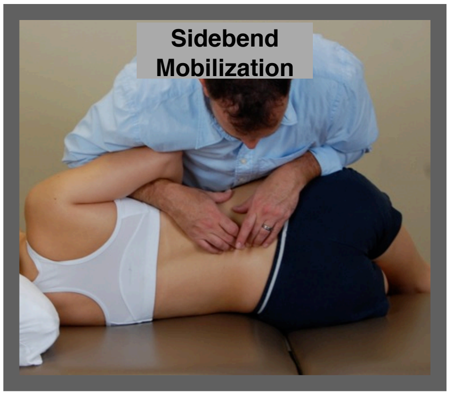
Sidebend Mobilization

Clinician Position:
Face the patient. Positioned high enough to have upper trunk over patient.
Technique Description:
Repeatedly bring your index fingers up and together and bring your thumbs out and away (book opening motion) in order to open up the foraminal space on the upward side of the patient.

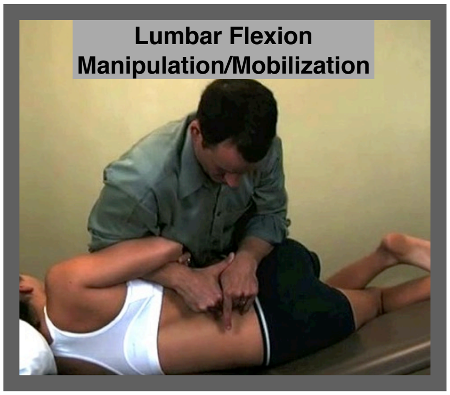
Lumbar Flexion Manipulation/Mobilization

Clinician Position:
Face the patient. Positioned high enough to have upper trunk over patient.
Technique Description:
Bias patient's lumbar spine into flexion. Flex the lumbar spine until movement is felt at intended segment. Apply thrust with distal arm into anterior and cephalad direction.

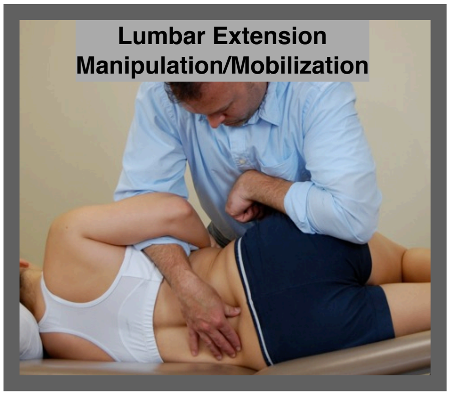
Lumbar Extension Manipulation/Mobilization

Clinician Position:
Face the patient. Positioned high enough to have upper trunk over patient.
Technique Description:
Bias patient's lumbar spine into extension. Take up all the motion, and then let out a little tension. Apply thrust with distal arm into anterior and cephalad direction.

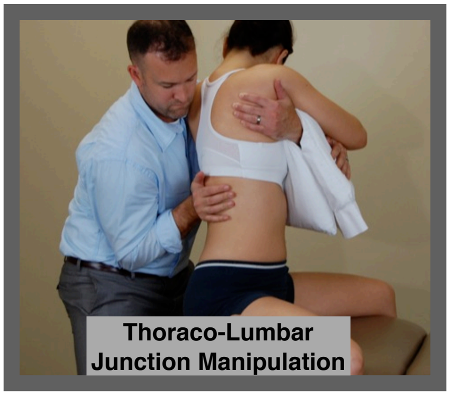
Thoraco-Lumbar Junction Manipulation

Clinician Position:
Stand behind the patient with the hypothenar part of the mobilizing hand on T-L Junction.
Technique Description:
Rotate until the slack is taken up in motion and then impart a high velocity thrust in an anterior and medial direction.

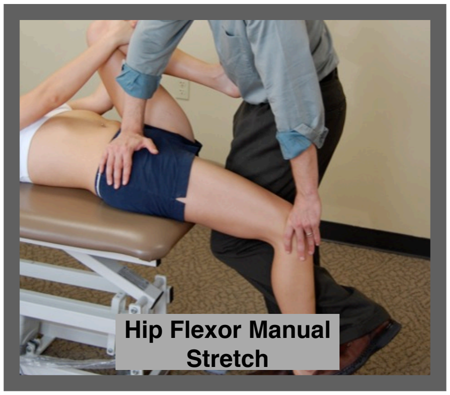
Hip Flexor Manual Stretch

Clinician Position:
Standing at the feet of the patient on the contralateral side.
Technique Description:
Iliopsoas: Provide downward force on femur and hold for 30 seconds.
Rectus Femoris: Introduce knee flexion
TFL: Introduce hip adduction and internal rotation

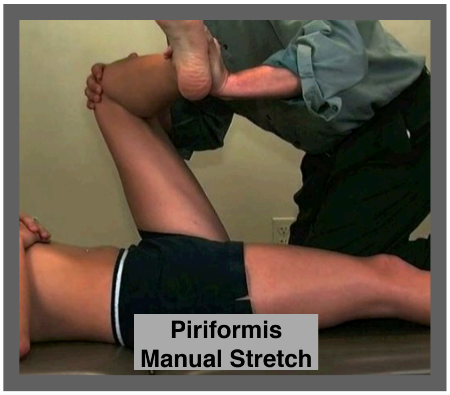
Piriformis Manual Stretch

Clinician Position:
Standing, facing cephalward on the side to be treated with your inside knee placed on the table.
Technique Description:
Bring patient's hip into into flexion and external rotation. Gently push further into flexion, external rotation, and adduction. Hold for 30 seconds
