# Supplementary material for: COMParative Early Treatment Effectiveness between physical therapy and usual care for low back pain (COMPETE): study protocol for a randomized controlled trial
Source: Trials. 2015 Sep 23;16:423. doi: 10.1186/s13063-015-0959-8 (PMC4581511; doi:10.1186/s13063-015-0959-8)
Supplement: Additional file 2: — Appendix 2. Treatment-based Classification Approach - Manual Therapy. Appendix 3. Treatment-based Classification Approach - Core Strengthening. Appendix 4. Treatment-based Classification Approach - Extension Oriented Treatment Approach. (PDF 792 kb) [file 13063_2015_959_MOESM2_ESM.zip › additional file 2/13063_2015_959_add3.pdf]

Core Stabilization/Strength Treatment Protocol

COMPETE Low Back Pain Trial

This following is the treatment protocol for core stabilization and strengthening program. A summary of the frequency of treatment is outlined below:

	Week 1	Week 2	Week 3	Week 4
**CORE Strengthening Exercises (8 sessions)**	2 sessions one on one	2 sessions group	2 sessions group	2 sessions group

Patients receiving this treatment will receive core strengthening exercises focused on improving motor control of the trunk stabilizing muscles (lumbar multifidus, transverse abdominus, rectus abdominus, etc). They will get a combination of individualized and group focus on strengthening progression exercises. Patients within the Manual Therapy or the EOTA treatment groups may transition to this treatment protocol based on how they improve after receiving their original respective treatments and clinician judgement. If they do, they will start out at the beginning of the program and finish the number of sessions that would be left in their four-week rehab program. Group sessions will have no more than 3 patients per therapist. Patient should also receive education as outlined below:

1. Education Component:
The clinician should review the education component on the patient's exercise handout. Key messages to emphasize should include:

- Frequency of the exercises in the home exercise program
- Encourage to perform activities even if there is some slight discomfort.
- Encourage patient to stay active
- Encourage patient to do abdominal drawing in maneuver (ADIM) with activities of daily living (ADL's) and activities

2) CORE Strength One on One Component:
These sessions will focus on appropriate contraction of the Transverse Abdominal (TrA) and Lumbar Multifidus (LM). The subject will be taught to contract these muscles during related ADL activity. Each session will last approximately 30 minutes.

3) CORE Strength Group Component:
These classes will put the subjects through a strengthening program focused on progressing and increasing the difficulty of core-related activities and tasks. Subjects will attend a class in a group setting (no more than 3 patients per class), which will include a warm-up and cool down and last approximately 30-45 minutes.

4) CORE Strength Home Exercise Program:
Exercises- The patient will perform pelvic tilts for mobility. The core strengthening exercises will be progressed as outlined in the Table below

**Activity**	**Progression**
1. TrA-DIM	Supine with knees bent - foot off table - knee extension
2. Bridging	Bilateral – hands on chest – foot 1" off table – leg extension
3. Sideplank	Side on knees – side on ankles – hip abduction
4. Quadruped	TrA-DIM – single arm – single leg – alternating arm/leg
