# Supplementary material for: COMParative Early Treatment Effectiveness between physical therapy and usual care for low back pain (COMPETE): study protocol for a randomized controlled trial
Source: Trials. 2015 Sep 23;16:423. doi: 10.1186/s13063-015-0959-8 (PMC4581511; doi:10.1186/s13063-015-0959-8)

Appendix B1

Extension-Oriented Treatment Protocol

COMPETE Low Back Pain Trial

This following is the treatment protocol for the Extension-Oriented Treatment Approach (EOTA) that includes mechanical lumbar traction plus reinforcing exercises. A summary of the frequency of treatment is outlined below:

	**Week 1**	**Week 2**	**Week 3**	**Week 4**
**EOTA + Mechanical traction (10 sessions)**	3 sessions: EOTA+ mechanical traction	2 sessions: EOTA+ mechanical traction	2 sessions: EOTA+ mechanical traction	1 sessions: EOTA+ mechanical traction

The treatment received by patients in this group will consist of mechanical traction in addition to exercise and mobilization to promote spinal extension. Patient should also receive education as outlined below:

1. Education Component:

You should review the education component on the patient's exercise handout. Key messages to emphasize should include:

- General instructions to discontinue any activities that cause their symptoms to peripheralize.
- Encourage to perform activities and maintain positions that centralize symptoms.
- Encourage patient to stay active
- How to maintain lumbar lordosis with ADL's (such as sitting)

2) Traction Component:

a) POSITION: Patients will be positioned in prone.

- Exceptions: If the patient has a flexion or lateral shift deformity that causes peripheralization of symptoms when attempting to lie flat, or if lying flat causes significant increased low back pain, the traction table position will initially be positioned in flexion and/or side bending as tolerated for subject comfort.
- If the patient has both a flexion and a lateral shift deformity, the table will be repositioned first to correct the lateral shift deformity and then to correct the flexion deformity. Achieving only partial correction of the deformity is acceptable, but the goal is to attempt as much correction as tolerated each session.
  "Tolerance" will be defined as the ability to achieve a neutral posture (or as close to neutral as possible) without peripheralization of symptoms or significant increase in low back pain
- HARNESS Set-Up: Position the lumbar harness about 1 inch superior to the pelvic crest. Secure the thoracic harness with about 1 inch overlap of the lumbar belt. Aggressively remove the slack from the belts which attach the harnesses to the table allowing the lumbar belt to slide into place taking hold of the pelvis

b) TYPE: Static mechanical traction
c) TIME: Traction will be applied for a total of 12 minutes, which consists of 10 minutes at the desired intensity, plus a 1-minute ramp up and 1-minute ramp down time.
d) FORCE: The intensity of the pull will be 40-60% of the patient's body weight, adjusted based on the patient's tolerance and symptom response.

Unlock the table and give patient the safety control switch.

Extension-Oriented Treatment Protocol

- After 3 minutes of traction treatment: if the table was initially positioned in flexion or side bending, the table will be repositioned as tolerated to achieve a neutral lumbar posture. Correct the lateral shift fist and progress to correcting any flexion component.

At the <u>end of the traction treatment</u>, patients will lie still for 2 minutes in prone.

- For patients who ended the traction treatment in a flat (neutral) position: tilt table up to 10° of extension as tolerated, and the patients will lie still in this position for an additional 2 minutes.
- For patients who did not end the traction treatment in a flat (neutral) position, no extension positioning will be attempted.

At the conclusion of the traction treatment, patients will be assisted to stand and walk.
Patient education: flexion, side bending and sitting positions are to be minimized as much as possible.

3) EOTA Component:

Exercises- The extension-oriented exercises will be progressed according to the goals outlined in the Table.

**Progression of Activity**	**Goal**
1. Prone lying	Able to tolerate for 5 minutes, no pillow
2. Prone lying on elbows	Able to tolerate for 5 minutes
3. Prone press-up exercise	3 sets of 10 reps, move to end-range extension
4. Repeated extension in standing	3 sets of 10 reps, move to end-range of extension

- If needed, patients will begin the program by lying prone on the table, using pillows as needed to accommodate patients unable to initially tolerate lying in prone (i.e. presence of an acute kyphosis). When they are able to tolerate this position for five minutes without the use of a pillow and no increase in symptoms, they will progress to lying prone on their elbows. When they are able to tolerate this position for five minutes without an increase in symptoms, they will perform 10 repetitions of the prone press-up exercise. Patients who are easily able to assume the first two positions will be allowed to progress directly to the prone press-up exercise on subsequent visits.
- The first 2-3 repetitions of the prone press-up exercise will be conducted to approximately half of the available range of extension. Patients will be instructed to gradually progress into more and more extension with each repetition so long as they do not experience a significant increase in symptoms, with the goal of moving to end range extension during the last repetition. At the end of the last repetition, patients will be asked to exhale, allowing their back to sag into maximal extension. If they tolerate the first set of prone press-ups, they will perform 2 additional sets of 10 repetitions each of the prone press-up exercise, emphasizing the end range of extension assisted by exhalation during the last repetition of each set.
- Once patients are able to tolerate 3 sets of 10 repetitions of the prone press-up exercise, they will perform 3 sets of 10 repetitions of repeated extension in standing by placing their hands in the "small of their back" with their feet shoulder width apart and lean

backwards as far as possible, without increasing their symptoms. Therapists will advise the patient to emphasize lumbar spine extension and to minimize extension occurring in the cervical spine.

- LATERAL SHIFT: all extension exercises may be performed with a lateral side-bending component if this facilitates centralization of symptoms.
- Patients will be instructed to monitor their symptoms and perform the exercises in a range of movement (i.e. beginning, middle, or end-range) that maximizes centralization of their symptoms and minimizes an increase in the intensity of symptoms.

HEP: Patients will be instructed to perform the highest level of EOTA activity they have achieved in the clinic every 4-5 hours on days the patients do not attend physical therapy.

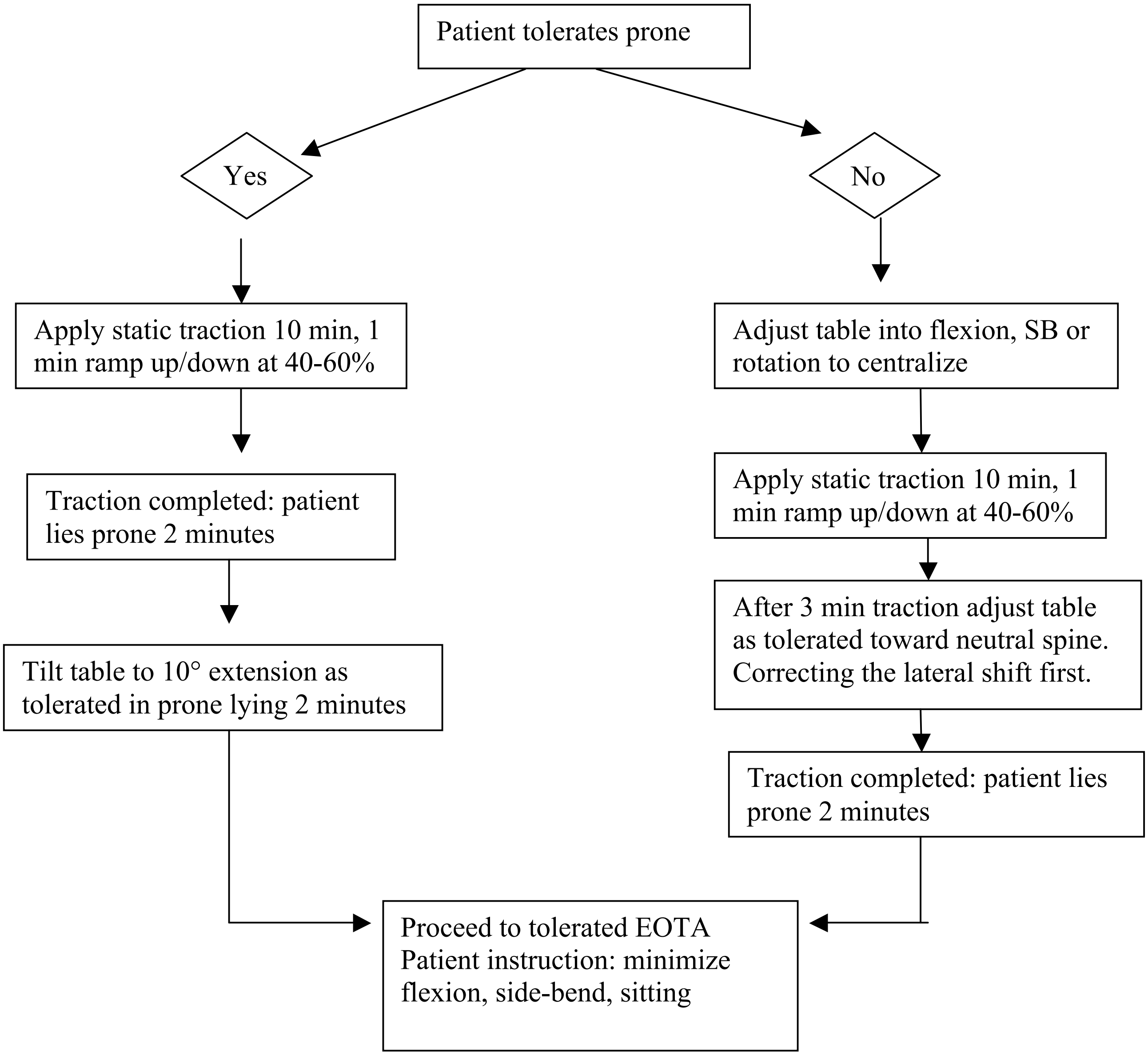

Supplement: Additional file 2: — Appendix 2. Treatment-based Classification Approach - Manual Therapy. Appendix 3. Treatment-based Classification Approach - Core Strengthening. Appendix 4. Treatment-based Classification Approach - Extension Oriented Treatment Approach. (PDF 792 kb) [file 13063_2015_959_MOESM2_ESM.zip › additional file 2/13063_2015_959_add4.pdf]
