# Supplementary material for: COMParative Early Treatment Effectiveness between physical therapy and usual care for low back pain (COMPETE): study protocol for a randomized controlled trial
Source: Trials. 2015 Sep 23;16:423. doi: 10.1186/s13063-015-0959-8 (PMC4581511; doi:10.1186/s13063-015-0959-8)
Supplement: Additional file 3: — Appendix 5. Home Exercise Program. (PDF 2337 kb) [file 13063_2015_959_MOESM3_ESM.pdf]

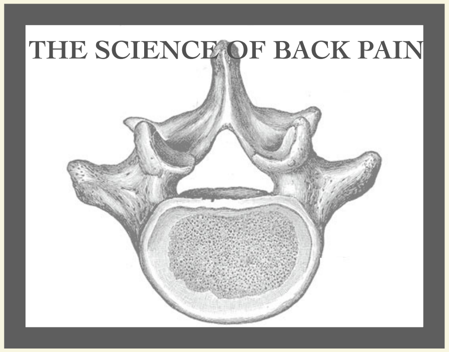

The Truth of About Low Back Pain

- Acute pain usually resolves in several days or a few weeks for the large majority of individuals
- A very minor percentage of low back pain ends up being a serious condition that requires a surgery. It is very rare. For those few conditions that occur rarely, the signs are relatively easy for your health care provider to identify.
- X-rays and MRI's are not often needed to determine how to manage your back pain
- Surgery is very rarely needed
- The majority of people can and should get moving early even if there is some pain
- Your spine is very resilient and strong. You may be surprised at how difficult it is to damage your spine.
- Even severe pain does not necessarily indicate likely injury or serious damage.
- A large majority of adults experience an episode of low back pain

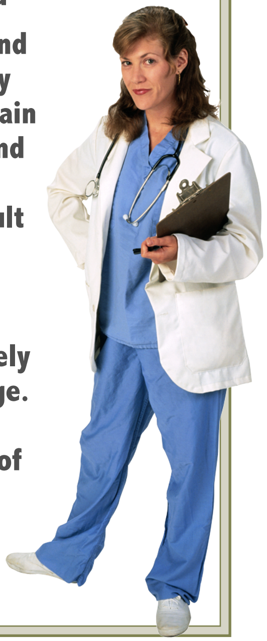

"THIS IS MORE THAN JUST A METHOD TO MANAGE YOUR LOW BACK PAIN, IT'S A **NEW WAY TO THINK** ABOUT YOUR BACK PAIN, BASED ON THE LATEST CURRENT SCIENCE OF BACK PAIN"

WHAT CAUSES BACK PAIN

- DISCS?
- JOINTS?
- MUSCLES?
- NERVES?
- SPINAL CORD?
- VERTEBRAE?

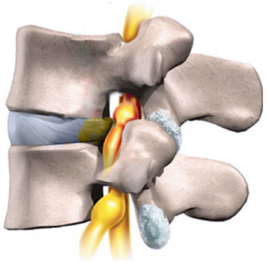

THE TRUTH IS THAT A SPECIFIC ANATOMICAL CAUSE CAN ONLY BE RELIABLY IDENTIFIED IN 10-15% OF ALL CASES OF LOW BACK PAIN. IT COULD BE A COMBINATION OF FACTORS, BUT KNOWING THE TRUE CAUSE WILL LIKELY NOT CHANGE YOUR OUTCOME

ULTIMATE GOAL
"GET UP AND GET MOVING"

LOW BACK PAIN

MANAGEMENT

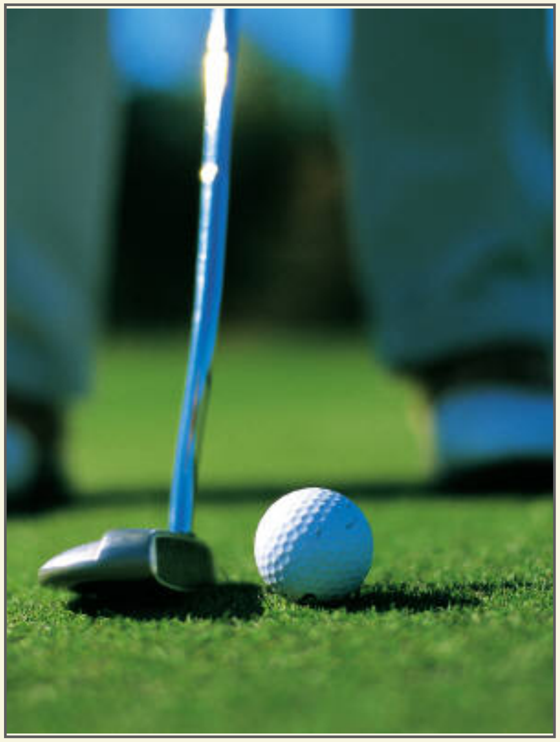

ANXIETY & STRESS

- Has been shown to aggravate and prolong low back pain
- Attitudes of fear and worry over your back have been shown to delay recovery

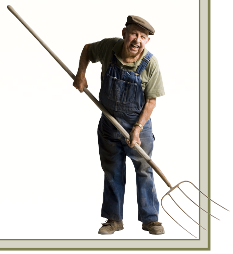

Activity Recommendations

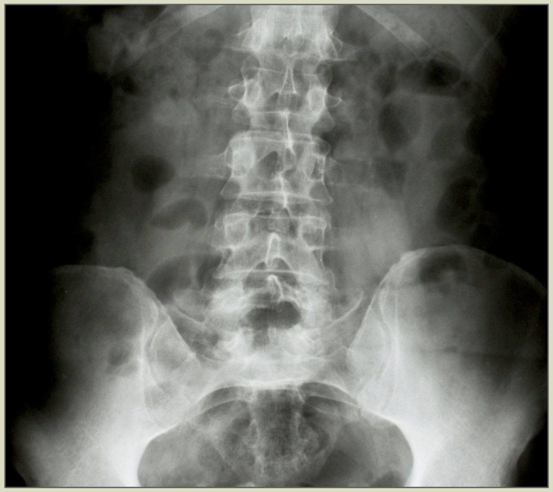

X-RAYS AND MRIs

You may think your doctor needs an X-ray or MRI to understand your the nature of your problem, however routine radiographs for low back pain are not recommended. The gonadal radiation received from one lumbar X-ray is equivalent to getting 1 chest X-ray daily for an entire year. Often the findings from the MRI will not tell your doctor information relevant to your treatment plan, or add to what they can determine from the clinical examination. Anywhere from 30-40% of individuals without low back pain have abnormal findings on MRI (degenerative discs or joints, herniated discs, annular tears, etc.). These finding are often not the best predictors of your prognosis.

WHEN SHOULD I SEE MY DOCTOR?

- Severe pain that gets worse for several weeks instead of better
- If you feel sick in addition to your back pain
- If your leg pain is as bad or worse then your back pain

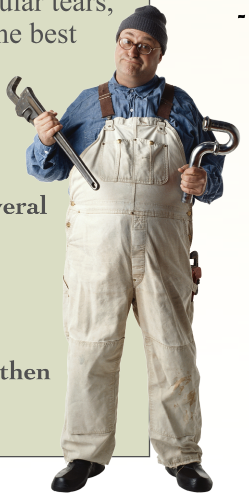

WHAT TO DO?

- Stay with your normal routine, and just move a little further and faster each day. Change the way you perform some of your activities if necessary, but don't stop doing those activities.
- Try to move around a few times every hour.
- Remember, even in the presence of pain, exercise is recommended.
- Think about your smoking habits and weight and how they may impact your recovery.
- Take frequent breaks from prolonged sitting
- Ice for the first 48 hours and then after that moist heat

WHAT CAN MY PROVIDER DO FOR ME?

- Rule out any serious diseases
- Suggest treatments and medications
- Provide advice for the best way to deal with the pain

WHAT I SHOULD NOT EXPECT FROM MY PROVIDER?

- A "quick fix" or "miracle cure"
- A prescription of bed rest or inactivity

SEEK A MEDICAL PROFESSIONAL IMMEDIATELY IF YOU HAVE:

- Pins and needles or severe weakness in BOTH legs
- Difficulty walking
- Pain that does not get better when move or change positions
- Pain that wakes you up at night
- Night sweats
- Difficulty passing or controlling urine

MEDICATIONS

If you feel that you need some pain medication you should always start with simple over the counter analgesics such as ibuprofen or aspirin (always check with your doctor before taking any medication). Commonly prescribed medications such as ibuprofen and muscle relaxers have shown to provide a slight benefit over placebo for both acute and chronic low back pain, but the benefit should not be expected beyond short term. Some of these medications can have side-effects. Your doctor may prescribe either depending on what they think may be best for you. Neither of these medications have been shown to provide long term relief.

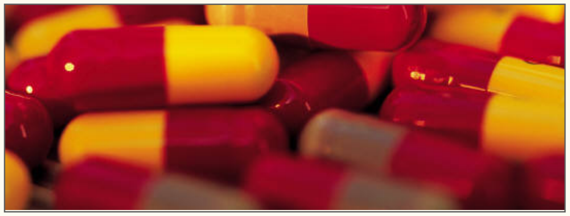

HARM WITH RESTING	BENEFITS OF ACTIVITY
DECONDITIONING	NATURAL ENDORPHINS FOR PAIN RELIEF
WEAKENING OF BONES	STRENGTHEN MUSCLE & BONES
LOSS OF FLEXIBILITY	PREVENT BACK STIFFNESS
SOCIAL WITHDRAWAL	STIMULATE BODY'S OWN ABILITY TO HEAL
DEPRESSION	

The American College of Physicians and the American Pain Society highly recommend that patients with nonspecific low back pain receive evidence-based information with advice to stay active, the expected course of their symptoms, and effective self-care options.
